# Use of CytoSorb in Traumatic Amputation of the Forearm and Severe Septic Shock

**DOI:** 10.1155/2017/8747616

**Published:** 2017-12-20

**Authors:** Heinz Steltzer, Alexander Grieb, Karim Mostafa, Reinhard Berger

**Affiliations:** ^1^AUVA-Unfallkrankenhaus Meidling, Department of Anesthesiology and Intensive Care Medicine, Vienna, Austria; ^2^Sigmund Freud Private University, Vienna, Austria

## Abstract

Severe trauma associated with later disability and mortality still constitutes a major health and socioeconomic problem throughout the world. While primary morbidity and mortality are mostly related to initial injuries and early complications, secondary lethality is strongly linked to the development of systemic inflammatory response syndrome, sepsis, and ultimately multiple organ dysfunction syndrome. We herein report on a 49-year-old male patient who was admitted to the hospital after a traumatic amputation of his right forearm that was cut off while working on a landfill. After initial treatment for shock, he received immediate replantation and was transferred to the ICU. Due to the anticipated risk of a complex infection, continuous renal replacement therapy in combination with CytoSorb was initiated. During the course of the combined treatment, a rapid improvement in hemodynamics was noticed, as well as a significant reduction of IL-6 and lactate levels. Despite a recurring septic episode and the necessity for amputation, the patient clinically stabilized and underwent complete recovery. The early treatment with a combination of CVVHDF and CytoSorb was accompanied by an attenuation of the systemic inflammatory reaction, which subsided without major or permanent organ damage, despite the impressive pathogen spectrum and the pronounced local damage.

## 1. Introduction

Severe trauma associated with later disability and mortality still constitutes a major health and socioeconomic problem throughout the world [1]. The traumatized patient presents with a series of posttraumatic complications secondary to the traumatic injury itself. While primary morbidity and mortality are mostly related to initial injuries (i.e., severe traumatic brain injury, hemorrhagic shock) and early complications (i.e., acidosis, coagulopathy, hypothermia, oxidative stress, metabolic disorders), secondary lethality is strongly linked to the development of systemic inflammatory response syndrome (SIRS), sepsis, and ultimately multiple organ dysfunction syndrome (MODS) [2]. The poor outcome of these patients is therefore directly related to the association of the aforementioned pathologies.

Several factors may lead to hemodynamic instability and insufficient tissue perfusion, necessitating fluid resuscitation and catecholamine support. Therefore, initial protection of circulation and avoidance of hypoperfusion, initially most often related to blood loss and hypovolemia, is critical in these patients. As blood pressure and heart rate prove partly unreliable to evaluate cardiac output in critically injured patients, increase in lactate levels may help identify a patient whose initially normal vital signs may disguise tissue hypoperfusion [3].

On the other hand, a plethora of cytokines play a role in the inflammatory response subsequent to critical injury. Particularly interleukin-6 (IL-6) appears to play an active role in the postinjury immune response. IL-6 is released in response to an inflammatory stimulus or tissue injury, acting locally and systemically to generate a multitude of physiologic responses. The release of IL-6 in response to traumatic injury mimics that to elective surgery, with IL-6 levels rising early and preceding acute phase protein expression, making it an attractive diagnostic but even more a therapeutic target in attempts to control hyperinflammation-associated organ dysfunction [4].

In this context, extracorporeal blood purification therapy using a recently introduced hemoadsorption device (CytoSorb) did show promising results in patients with SIRS and sepsis by attenuating the cytokine-driven overwhelming inflammatory response, improving hemodynamic stability as well as other clinically relevant parameters [5–7].

We herein report on a patient who was successfully treated with CytoSorb after a traumatic amputation of his right forearm with subsequent development of severe septic shock due to infection with multiresistant pathogens. Informed consent for data analysis and publication was obtained from the patient.

## 2. Case Presentation

A 49-year-old male patient was admitted to the hospital via helicopter transport after a traumatic amputation of his right forearm. Earlier, while working on a landfill cleaning surfaces with a high pressure cleaner, the air pressure tube caught his arm and his right forearm was cut off at the elbow joint. The amputate was not damaged macroscopically; however a wide spectrum of various aerobic and anaerobic pathogens was detected in the wound later on, many of which were multiresistant.

On admission, the patient was treated for shock (RR = 69/55 HR 99 and two hours later at RR 71/68, HR 110), followed by X-ray examination and immediate replantation (operation time of approx. 8.5 hours). After successful surgery and a resulting well-perfused transplant, the patient was postoperatively transferred to the intensive care unit intubated, ventilated, and catecholamine-dependent (norepinephrine 0.41 *μ*g/kg/min) with a mean arterial pressure of 65 mmHg. We noticed development of lactic acidosis (3.9 mmol/l) and in the further course a sharp increase in inflammation-relevant parameters (leukocytes 18.700/*μ*l, CRP 13.5 mg/dl, PCT 0.88 ng/ml, IL-6 > 5000 pg/ml). Continuous renal replacement therapy (CRRT) was started due to anuria postshock and the onset of acute renal failure. Detected pathogens included* Aeromonas hydrophila*, an enterotoxin-producing bacterium being endemic in the American tropics;* Stenotrophomonas maltophilia*, a multiresistant nosocomial pathogen detected in dialysis fluid; and* Clostridium subterminale*, which has been described in the medical literature only in nine case reports as being pathogenic. Antibiotic therapy (normal dosages) with sultamicillin 3 × 3 g (first 4 days), piperacillin/tazobactam 4.5 g (for 2 days), clarithromycin (500 mg 2 × 1), and meropenem (3 × 2 g) for 10 days was initiated and hydrocortisone (20 mg/h) plus three red packed blood cells were administered immediately. Due to the anticipated risk of a complex infection because of the location of the accident (landfill), CytoSorb was additionally installed into the CRRT circuit. A total of 6 consecutive treatments with CytoSorb over 3 days with therapy intervals of 12 hours each were carried out. Therapy sessions were partly interrupted by surgical procedures. The CytoSorb treatment was performed in combination with standard continuous hemodiafiltration (CVVHDF, Fresenius Multifiltrate, Fresenius Medical Care AG, Bad Homburg, Germany) using regional citrate anticoagulation and blood flow rates of 100 ml/min. The CytoSorb adsorber was installed in prehemofilter position.

**Figure 1:**
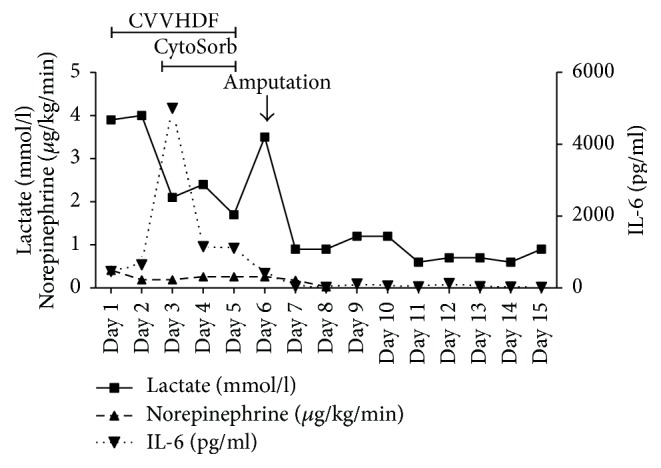
Plasma concentrations of IL-6 and lactate and the need of noradrenalin during the course of the eight treatment sessions. Day 1 represents start of treatment directly after postoperative transfer to ICU.

During the course of the combined CVVHDF-CytoSorb treatment we measured demand for catecholamines, inflammatory parameters (IL-6), and lactate levels (Figure 1). We noticed a clear and more importantly rapid improvement in hemodynamics with reduction of norepinephrine dosages from 0.41 down to 0.26 *μ*g/kg/min already within the very first treatment. Norepinephrine infusion rates had to be adjusted at a low scale during the subsequent treatment sessions, which was most likely due to the ongoing infection with multiresistant pathogens and the preceding surgical procedure. Furthermore, we observed a significant reduction of inflammatory parameters, in particular of IL-6, which decreased from >5000 pg/ml to 43 pg/ml one day after the last CytoSorb therapy session.

Initial peak levels of lactate (4 mmol/l) were progressively declining during the next treatments and reached normal values on the third day of CytoSorb treatment. Importantly, after cessation of CytoSorb treatment, we noticed a rebounce of plasma lactate levels in the context of an acute infection and necrosis of the amputate necessitating removal of necrotic tissue and ultimately amputation of the forearm. After amputation, the patient continuously stabilized and improved. Total CRRT time was 5 days, and pressure-controlled ventilation was carried out for 2 days (directly postoperative), was then changed to BiPAP and CPAP ventilation on the following 3 days, and completely stopped on day 5 together with CRRT and CytoSorb. Daily surgical wound care with disinfection and removal of necrotic tissue were performed in the further course. 18 days after initial admission, the patient was transferred to the normal trauma-surgical ward and underwent complete recovery with later adaptation of a robotic prosthesis.

## 3. Discussion

In the present case report, we treated a critically injured patient after traumatic amputation and subsequent development of severe septic shock with a combination of CVVHDF plus hemoadsorption. Treatment was associated with a rapid hemodynamic stabilization and a decrease in IL-6 as well as blood lactate levels.

One of the most prominent observations in our patient was the promptness in which hemodynamic stabilization occurred. From the data available on CytoSorb in critically ill patients in the context of sepsis and postcardiac surgery SIRS, we found clear conformance as to hemodynamic stabilization with a quick reduction in catecholamine dosages being one of the main effects to be expected from the application of the device [5–7].

In our patient, IL-6 plasma levels could be reduced drastically during the course of the combined treatments. There is evidence that the magnitude of IL-6 elevation after mechanical trauma appears to correlate with the extent of trauma severity, the risk of postinjury complications, and even adverse outcome [4, 8]. Moreover, a study conducted in a cohort of severe trauma patients by Sousa and coworkers showed that several cytokines were associated with outcomes, especially IL-6 and IL-10 at 72 h with MODS and death [9]. Likewise, Guisasola et al. found that patients with MODS had higher plasma levels of IL-6 and TNF-*α* which therefore suggests a potential role of these mediators as early predictive markers for systemic inflammatory response and clinical complications to stratify patients as to which therapeutic intervention they should receive [10]. Of note, data even suggest IL-6 as a helpful indicator in deciding which primary operation to perform (i.e., external fixator or intramedullary nail) and determining the optimal time for secondary surgery [11].

We monitored a progressive and rapid decline in lactate levels in our patient. With postoperative levels of 3.9–4 mmol/l the patient was still not in full-blown lactic acidosis. However, elevated lactate at this level points towards microcirculatory failure and hypoperfusion of vital organs and requires serious attention. Importantly, studies show that the degree of lactate elevation and the rate of lactate clearance strongly correlate with the risk of MODS and mortality after traumatic injury [12]. As initial lactate levels have been found to be significantly higher in trauma nonsurvivors compared to survivors, lactate levels and its clearance could potentially serve as an endpoint to guide resuscitation [13]. For example, Aslar and colleagues reported a calculated specificity of 86% and sensitivity of 84% for patients with torso trauma and a lactate level > 4 mmol/L to die [14].

After receiving and reviewing the pathogenic spectrum, we did anticipate a high risk of complex infection. Pathogens were rather uncommon and did pose a challenge concerning the right antibiotic therapy to administer.

In conclusion, this case is, to the best of our knowledge, the first published report on the clinical application of CytoSorb hemoadsorption in a patient with severe trauma and septic shock. The early treatment with a combination of CVVHDF and CytoSorb was accompanied by an attenuation of the systemic inflammatory reaction, which subsided without major or permanent organ damage, despite the impressive pathogen spectrum and the pronounced local damage. Treatment was safe and well tolerated. CytoSorb appears to possibly represent a promising adjunctive therapy to treat critically injured patients; however large, controlled studies are urgently required to determine the true benefit of this treatment in this subset of patients.

## References

[1] Peden M. M. K. S. G. (2002). *The Injury Chart Book: A Graphical Overview of The Global Burden of Injuries*.

[2] Rogobete A. F., Sandesc D., Papurica M. (2017). The influence of metabolic imbalances and oxidative stress on the outcome of critically ill polytrauma patients: A review. *Burns & Trauma*.

[3] Wo C. C. J., Shoemaker W. C., Appel P. L., Bishop M. H., Kram H. B., Hardin E. (1993). Unreliability of blood pressure and heart rate to evaluate cardiac output in emergency resuscitation and critical illness. *Critical Care Medicine*.

[4] Biffl W. L., Moore E. E., Moore F. A., Peterson V. M. (1996). Interleukin-6 in the injured patient: marker of injury or mediator of inflammation?. *Annals of Surgery*.

[5] Träger K., Skrabal C., Fischer G. (2017). Hemoadsorption treatment of patients with acute infective endocarditis during surgery with cardiopulmonary bypass—A case series. *The International Journal of Artificial Organs*.

[6] Träger K., Fritzler D., Fischer G. (2016). Treatment of post-cardiopulmonary bypass SIRS by hemoadsorption: A case series. *The International Journal of Artificial Organs*.

[7] Kogelmann K., Jarczak D., Scheller M., Drüner M. (2017). Hemoadsorption by CytoSorb in septic patients: a case series. *Critical Care*.

[8] Jawa R. S., Anillo S., Huntoon K., Baumann H., Kulaylat M. (2011). Interleukin-6 in surgery, trauma, and critical care Part II: Clinical implications. *Journal of Intensive Care Medicine*.

[9] Sousa A., Raposo F., Fonseca S. (2015). Measurement of cytokines and adhesion molecules in the first 72 hours after severe trauma: association with severity and outcome. *Disease Markers*.

[10] Guisasola M. C., Ortiz A., Chana F., Alonso B., Vaquero J. (2015). Early inflammatory response in polytraumatized patients: Cytokines and heat shock proteins. A pilot study. *Orthopaedics & Traumatology: Surgery & Research*.

[11] Van Griensven M. (2014). Cytokines as biomarkers in polytraumatized patients. *Der Unfallchirurg*.

[12] Manikis P., Jankowski S., Zhang H., Kahn R. J., Vincent J.-L. (1995). Correlation of serial blood lactate levels to organ failure and mortality after trauma. *The American Journal of Emergency Medicine*.

[13] Odom S. R., Howell M. D., Silva G. S. (2013). Lactate clearance as a predictor of mortality in trauma patients. *Journal of Trauma and Acute Care Surgery*.

[14] Aslar A. K., Kuzu M. A., Elhan A. H., Tanik A., Hengirmen S. (2004). Admission lactate level and the APACHE II score are the most useful predictors of prognosis following torso trauma. *Injury*.

